# 3-(4-Meth­oxy­phen­yl)-5-methylisoxazole-4-carb­oxy­lic acid

**DOI:** 10.1107/S1600536813004029

**Published:** 2013-02-16

**Authors:** Chandra, K. Raghu, N. Srikantamurthy, K. B. Umesha, K. Palani, M. Mahendra

**Affiliations:** a Department of Studies in Physics, Manasagangotri, University of Mysore, Mysore 570 006, India; b Department of Chemistry, Yuvaraja’s College, University of Mysore, Mysore 570 005, India; c SER-CAT, APS, Argonne National Laboratory, Argonne, IL 60439, USA

## Abstract

In the title compound, C_12_H_11_NO_4_, the dihedral angle between the benzene and isoxazole rings is 42.52 (8)°. The carb­oxy­lic acid group is close to being coplanar with the isoxazole ring [dihedral angle = 5.3 (2)°]. In the crystal, inversion dimers linked by pairs of O—H⋯O hydrogen bonds generate *R*_2_^2^(8) loops.

## Related literature

For the biological and pharmaceutical properties of isoxazoles, see: Changtam *et al.* (2010 ▶); Eddington *et al.* (2002 ▶); Kozikowski *et al.* (2008 ▶); Lee *et al.* (2009 ▶); Panda *et al.* (2009 ▶); Shin *et al.* (2005 ▶). For the agrochemical importance of isoxazoles, see: Pinho e Melo (2005 ▶7). For a related structure, see: Wolf *et al.* (1995 ▶).
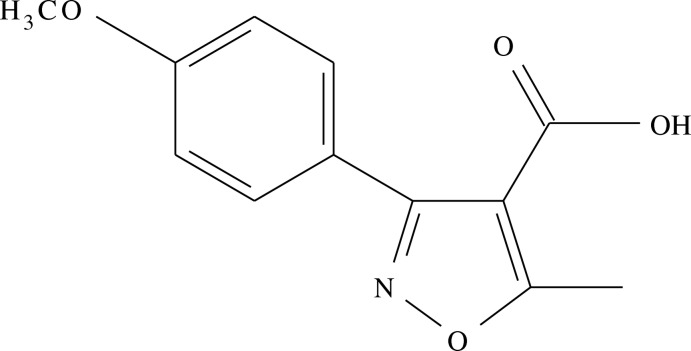


## Experimental

### 

#### Crystal data

C_12_H_11_NO_4_
*M* *_r_* = 233.22
Monoclinic, 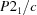

*a* = 6.4147 (2) Å
*b* = 14.6321 (6) Å
*c* = 11.9911 (5) Å
β = 97.220 (2)°
*V* = 1116.57 (7) Å^3^
*Z* = 4
Mo *K*α radiation
μ = 0.11 mm^−1^
*T* = 296 K
0.20 × 0.15 × 0.10 mm

#### Data collection

Bruker APEXII CCD diffractometer
Absorption correction: multi-scan (*SADABS*; Bruker, 2009 ▶) *T*_min_ = 0.979, *T*_max_ = 0.989
11200 measured reflections
2811 independent reflections
2083 reflections with *I* > 2σ(*I*)
*R*_int_ = 0.031

#### Refinement

*R*[*F* ^2^ > 2σ(*F* ^2^)] = 0.045
*wR*(*F* ^2^) = 0.125
*S* = 1.04
2811 reflections
156 parameters
H-atom parameters constrained
Δρ_max_ = 0.23 e Å^−3^
Δρ_min_ = −0.22 e Å^−3^

### 

Data collection: *APEX2* (Bruker, 2009 ▶); cell refinement: *SAINT* (Bruker, 2009 ▶); data reduction: *SAINT*; program(s) used to solve structure: *SHELXS97* (Sheldrick, 2008 ▶); program(s) used to refine structure: *SHELXL97* (Sheldrick, 2008 ▶); molecular graphics: *PLATON* (Spek, 2009 ▶); software used to prepare material for publication: *SHELXL97*.

## Supplementary Material

Crystal structure: contains datablock(s) global, I. DOI: 10.1107/S1600536813004029/hb7037sup1.cif

Structure factors: contains datablock(s) I. DOI: 10.1107/S1600536813004029/hb7037Isup2.hkl

Supplementary material file. DOI: 10.1107/S1600536813004029/hb7037Isup3.cml

Additional supplementary materials:  crystallographic information; 3D view; checkCIF report

## Figures and Tables

**Table 1:** Hydrogen-bond geometry (Å, °)

*D*—H⋯*A*	*D*—H	H⋯*A*	*D*⋯*A*	*D*—H⋯*A*
O17—H17⋯O16^i^	0.82	1.79	2.6034 (16)	173

Symmetry code: (i) 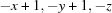.

**Fig. 1.**
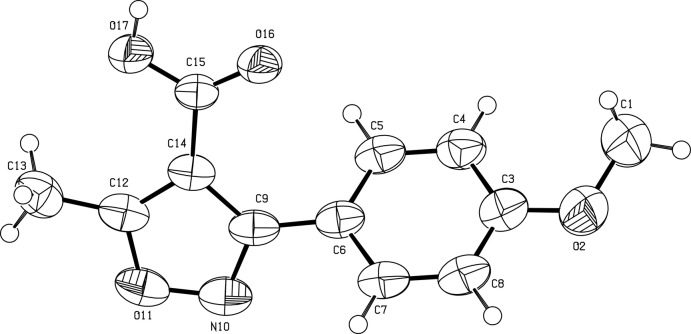
Perspective diagram of the molecule with 50% probability displacement ellipsoids.

**Fig. 2.**
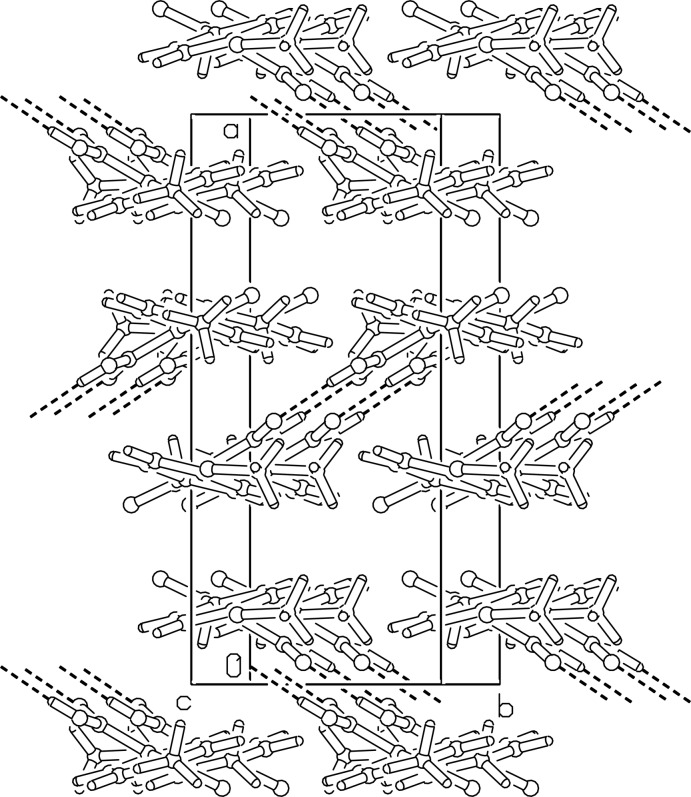
Packing diagram of the molecule viewed down the [010].

## supplementary crystallographic information

### Comment

Isoxazoles are five-membered heterocyclic ring structure with one oxygen atom and one nitrogen atom at adjacent positions. Isoxazoles have been widely used as a key building block for biological and pharmaceutical agents such as antiviral (Lee *et al.*, 2009), anti-mycobacterial (Changtam *et al.*, 2010), anti-tumor agents (Kozikowski *et al.*, 2008), anticonvulsant (Eddington *et al.*, 2002), antibacterial (Panda *et al.*, 2009) and anti-HIV activities (Shin *et al.*, 2005). In addition, derivatives of isoxazoles have been also studied as potential agrochemical properties including herbicidal and soil fungicidal activities; thus they have been used as pesticides and insecticides (Pinho e Melo, 2005). With this extensive backround of isoxazole derivatives, we have synthesized the title compound to study its crystal structure. Its potential antitumor activity against the aurora kinase enzyme will be described later.

In the molecular structure of the title compound (Fig. 1), the dihedral angle between the phenyl ring (C3/C4/C5/C6/C7/C8) and isoxazole ring (C9/N10/O11/C12/C14) is 42.52 (8)°. The isoxazole moiety is in *syn-periplanar* conformation with respect to the phenyl ring, as indicated by the torsion angle value of 0.60 (16)°. The carboxylic acid group of the isoxazole ring is nearly in the same plane (torsion angle = -5.3 (2)°) The bond lengths and angles agree with the observed values and are comparable to a related structure (Wolf *et al.*, 1995). The packing diagram of the molecules viewed down the *b* axis exhibits inversion dimers (Fig. 2).

### Experimental

A mixture of 4-methoxybenzaldehyde oxime (1 g, 0.0066 mmol), ethyl acetoacetate (1.7219 g, 0.0132 mmol), and chloramine-T trihydrate (1.8591 g, 0.0066 mmol) was warmed on a water bath for 3 h. After the reaction, it was cooled to room temperature. The sodium chloride formed in the reaction mixture was filtered off and washed with ethanol. The combined filtrate and washings were evaporated in vacuum. The residual part was extracted into ether (25 ml), washed successively with water (25 ml), 10% sodium hydroxide (25 ml) and saturated brine solution (10 ml). The organic layer was dried over anhydrous sodium sulfate. Evaporation of the solvent yielded the white solid. The isoxazole ester thus formed was refluxed with 10% NaOH for 4 hr. After the reaction, the reaction mass was acidified with dil. HCl to get title compound.

### Refinement

H atoms were placed at idealized positions and allowed to ride on their parent atoms with C–H distances in the range of 0.93 to 0.96 Å; *U*_iso_(H) = 1.2*U*_eq_(carrier atom) for all H atoms.

### Crystal data

**Table:**

C_12_H_11_NO_4_	*F*(000) = 488
*M**_r_* = 233.22	*D*_x_ = 1.387 Mg m^−^^3^
Monoclinic, *P*2_1_/*c*	Mo *K*α radiation, λ = 0.71073 Å
Hall symbol: -P 2ybc	Cell parameters from 2811 reflections
*a* = 6.4147 (2) Å	θ = 2.2–28.5°
*b* = 14.6321 (6) Å	µ = 0.11 mm^−^^1^
*c* = 11.9911 (5) Å	*T* = 296 K
β = 97.220 (2)°	Block, yellow
*V* = 1116.57 (7) Å^3^	0.20 × 0.15 × 0.10 mm
*Z* = 4	

### Data collection

**Table:**

Bruker APEXII CCD diffractometer	2083 reflections with *I* > 2σ(*I*)
ω and φ scans	*R*_int_ = 0.031
Absorption correction: multi-scan (*SADABS*; Bruker, 2009)	θ_max_ = 28.5°, θ_min_ = 2.2°
*T*_min_ = 0.979, *T*_max_ = 0.989	*h* = −8→8
11200 measured reflections	*k* = −19→19
2811 independent reflections	*l* = −16→15

### Refinement

**Table:**

Refinement on *F*^2^	Primary atom site location: structure-invariant direct methods
Least-squares matrix: full	Secondary atom site location: difference Fourier map
*R*[*F*^2^ > 2σ(*F*^2^)] = 0.045	Hydrogen site location: inferred from neighbouring sites
*wR*(*F*^2^) = 0.125	H-atom parameters constrained
*S* = 1.04	*w* = 1/[σ^2^(*F*_o_^2^) + (0.0516*P*)^2^ + 0.2571*P*] where *P* = (*F*_o_^2^ + 2*F*_c_^2^)/3
2811 reflections	(Δ/σ)_max_ < 0.001
156 parameters	Δρ_max_ = 0.23 e Å^−^^3^
0 restraints	Δρ_min_ = −0.22 e Å^−^^3^

### Special details

**Table:**

Geometry. Bond distances, angles *etc*. have been calculated using the rounded fractional coordinates. All su's are estimated from the variances of the (full) variance-covariance matrix. The cell e.s.d.'s are taken into account in the estimation of distances, angles and torsion angles
Refinement. Refinement on *F*^2^ for ALL reflections except those flagged by the user for potential systematic errors. Weighted *R*-factors *wR* and all goodnesses of fit *S* are based on *F*^2^, conventional *R*-factors *R* are based on *F*, with *F* set to zero for negative *F*^2^. The observed criterion of *F*^2^ > σ(*F*^2^) is used only for calculating -*R*-factor-obs *etc*. and is not relevant to the choice of reflections for refinement. *R*-factors based on *F*^2^ are statistically about twice as large as those based on *F*, and *R*-factors based on ALL data will be even larger.

### Fractional atomic coordinates and isotropic or equivalent isotropic displacement parameters (Å^2^)

**Table:**

	*x*	*y*	*z*	*U*_iso_*/*U*_eq_	
O2	0.2009 (2)	0.37793 (11)	0.57223 (11)	0.0848 (6)	
O11	−0.25133 (16)	0.31776 (8)	−0.04335 (11)	0.0656 (4)	
O16	0.35249 (16)	0.46156 (8)	0.09298 (9)	0.0573 (3)	
O17	0.30828 (17)	0.44090 (8)	−0.09213 (9)	0.0639 (4)	
N10	−0.2230 (2)	0.31499 (10)	0.07570 (13)	0.0649 (5)	
C1	0.4132 (4)	0.37406 (19)	0.62109 (19)	0.0930 (9)	
C3	0.1563 (3)	0.37111 (12)	0.45858 (15)	0.0627 (6)	
C4	0.2992 (3)	0.34453 (12)	0.38735 (15)	0.0632 (6)	
C5	0.2364 (2)	0.33990 (11)	0.27343 (15)	0.0595 (5)	
C6	0.0325 (2)	0.36064 (9)	0.22778 (14)	0.0530 (5)	
C7	−0.1095 (3)	0.38676 (11)	0.30087 (16)	0.0610 (6)	
C8	−0.0479 (3)	0.39210 (13)	0.41410 (17)	0.0673 (6)	
C9	−0.0393 (2)	0.35217 (9)	0.10713 (14)	0.0522 (5)	
C12	−0.0825 (2)	0.35580 (9)	−0.07996 (14)	0.0534 (5)	
C13	−0.0912 (3)	0.36697 (12)	−0.20259 (15)	0.0668 (6)	
C14	0.0574 (2)	0.37919 (9)	0.01037 (13)	0.0482 (4)	
C15	0.2528 (2)	0.42983 (9)	0.00688 (12)	0.0456 (4)	
H1A	0.47240	0.31620	0.60400	0.1390*	
H1B	0.42050	0.38080	0.70110	0.1390*	
H1C	0.49070	0.42250	0.59130	0.1390*	
H4	0.43650	0.32990	0.41620	0.0760*	
H5	0.33320	0.32240	0.22590	0.0710*	
H7	−0.24740	0.40070	0.27230	0.0730*	
H8	−0.14420	0.41000	0.46170	0.0810*	
H13A	−0.19650	0.32700	−0.23990	0.1000*	
H13B	0.04310	0.35200	−0.22520	0.1000*	
H13C	−0.12590	0.42910	−0.22260	0.1000*	
H17	0.41870	0.46970	−0.08720	0.0960*	

### Atomic displacement parameters (Å^2^)

**Table:**

	*U*^11^	*U*^22^	*U*^33^	*U*^12^	*U*^13^	*U*^23^
O2	0.0740 (9)	0.1114 (11)	0.0710 (9)	0.0108 (8)	0.0169 (7)	0.0006 (7)
O11	0.0456 (6)	0.0581 (6)	0.0920 (9)	−0.0111 (5)	0.0041 (6)	−0.0073 (6)
O16	0.0490 (6)	0.0642 (6)	0.0598 (6)	−0.0145 (5)	0.0111 (5)	−0.0062 (5)
O17	0.0533 (6)	0.0795 (8)	0.0600 (7)	−0.0195 (5)	0.0115 (5)	−0.0047 (5)
N10	0.0499 (7)	0.0596 (8)	0.0863 (10)	−0.0112 (6)	0.0135 (7)	0.0006 (7)
C1	0.0791 (14)	0.1213 (19)	0.0774 (13)	0.0088 (13)	0.0054 (11)	0.0043 (12)
C3	0.0609 (10)	0.0593 (9)	0.0704 (11)	0.0010 (7)	0.0178 (8)	0.0052 (8)
C4	0.0501 (9)	0.0661 (10)	0.0749 (11)	0.0067 (7)	0.0133 (8)	0.0095 (8)
C5	0.0490 (8)	0.0571 (9)	0.0759 (11)	0.0060 (7)	0.0211 (7)	0.0057 (7)
C6	0.0474 (8)	0.0423 (7)	0.0716 (10)	−0.0025 (6)	0.0164 (7)	0.0062 (6)
C7	0.0448 (8)	0.0596 (9)	0.0810 (12)	0.0012 (6)	0.0177 (7)	0.0056 (8)
C8	0.0557 (9)	0.0705 (11)	0.0805 (12)	0.0050 (8)	0.0274 (8)	0.0019 (9)
C9	0.0408 (7)	0.0392 (7)	0.0783 (10)	−0.0009 (5)	0.0138 (7)	0.0021 (6)
C12	0.0421 (7)	0.0393 (7)	0.0786 (10)	−0.0005 (5)	0.0067 (7)	−0.0073 (6)
C13	0.0615 (10)	0.0635 (10)	0.0727 (11)	−0.0017 (8)	−0.0017 (8)	−0.0166 (8)
C14	0.0398 (7)	0.0375 (6)	0.0679 (9)	0.0006 (5)	0.0086 (6)	−0.0021 (6)
C15	0.0388 (7)	0.0410 (6)	0.0576 (8)	0.0012 (5)	0.0089 (6)	−0.0011 (6)

### Geometric parameters (Å, º)

**Table:**

O2—C1	1.414 (3)	C9—C14	1.438 (2)
O2—C3	1.361 (2)	C12—C14	1.361 (2)
O11—N10	1.417 (2)	C12—C13	1.474 (2)
O11—C12	1.3397 (17)	C14—C15	1.4612 (18)
O16—C15	1.2347 (18)	C1—H1A	0.9600
O17—C15	1.2918 (18)	C1—H1B	0.9600
O17—H17	0.8200	C1—H1C	0.9600
N10—C9	1.3096 (19)	C4—H4	0.9300
C3—C4	1.385 (3)	C5—H5	0.9300
C3—C8	1.385 (3)	C7—H7	0.9300
C4—C5	1.376 (3)	C8—H8	0.9300
C5—C6	1.387 (2)	C13—H13A	0.9600
C6—C9	1.467 (2)	C13—H13B	0.9600
C6—C7	1.395 (2)	C13—H13C	0.9600
C7—C8	1.368 (3)		
			
C1—O2—C3	118.84 (16)	O16—C15—C14	121.52 (13)
N10—O11—C12	109.60 (12)	O17—C15—C14	115.23 (13)
C15—O17—H17	109.00	O16—C15—O17	123.22 (13)
O11—N10—C9	105.96 (13)	O2—C1—H1A	109.00
O2—C3—C8	116.04 (17)	O2—C1—H1B	109.00
C4—C3—C8	119.47 (17)	O2—C1—H1C	109.00
O2—C3—C4	124.49 (17)	H1A—C1—H1B	109.00
C3—C4—C5	119.48 (17)	H1A—C1—H1C	110.00
C4—C5—C6	121.66 (15)	H1B—C1—H1C	109.00
C5—C6—C7	118.04 (16)	C3—C4—H4	120.00
C5—C6—C9	122.30 (13)	C5—C4—H4	120.00
C7—C6—C9	119.60 (13)	C4—C5—H5	119.00
C6—C7—C8	120.63 (17)	C6—C5—H5	119.00
C3—C8—C7	120.71 (18)	C6—C7—H7	120.00
N10—C9—C6	118.58 (14)	C8—C7—H7	120.00
N10—C9—C14	110.23 (14)	C3—C8—H8	120.00
C6—C9—C14	131.18 (12)	C7—C8—H8	120.00
O11—C12—C13	116.25 (14)	C12—C13—H13A	109.00
O11—C12—C14	108.85 (14)	C12—C13—H13B	109.00
C13—C12—C14	134.83 (14)	C12—C13—H13C	109.00
C9—C14—C15	128.39 (13)	H13A—C13—H13B	109.00
C12—C14—C15	125.95 (14)	H13A—C13—H13C	109.00
C9—C14—C12	105.36 (12)	H13B—C13—H13C	109.00
			
C1—O2—C3—C4	12.0 (3)	C7—C6—C9—C14	138.29 (16)
C1—O2—C3—C8	−168.49 (19)	C5—C6—C7—C8	0.4 (2)
C12—O11—N10—C9	0.60 (16)	C9—C6—C7—C8	177.77 (15)
N10—O11—C12—C13	−177.70 (13)	C6—C7—C8—C3	−0.4 (3)
N10—O11—C12—C14	−0.41 (15)	N10—C9—C14—C12	0.32 (16)
O11—N10—C9—C14	−0.55 (16)	N10—C9—C14—C15	174.29 (13)
O11—N10—C9—C6	178.83 (11)	C6—C9—C14—C12	−178.95 (14)
O2—C3—C8—C7	−179.51 (17)	C6—C9—C14—C15	−5.0 (2)
C4—C3—C8—C7	0.1 (3)	O11—C12—C14—C9	0.07 (14)
C8—C3—C4—C5	0.4 (3)	O11—C12—C14—C15	−174.09 (12)
O2—C3—C4—C5	179.90 (17)	C13—C12—C14—C9	176.64 (16)
C3—C4—C5—C6	−0.4 (3)	C13—C12—C14—C15	2.5 (3)
C4—C5—C6—C9	−177.26 (14)	C9—C14—C15—O16	−5.3 (2)
C4—C5—C6—C7	0.1 (2)	C9—C14—C15—O17	176.71 (13)
C5—C6—C9—N10	136.35 (15)	C12—C14—C15—O16	167.52 (14)
C5—C6—C9—C14	−44.4 (2)	C12—C14—C15—O17	−10.5 (2)
C7—C6—C9—N10	−40.94 (19)		

### Hydrogen-bond geometry (Å, º)

**Table:**

*D*—H···*A*	*D*—H	H···*A*	*D*···*A*	*D*—H···*A*
O17—H17···O16^i^	0.82	1.79	2.6034 (16)	173

Symmetry code: (i) −*x*+1, −*y*+1, −*z*.
